# Screening of preservatives and evaluation of sterilized cellulose nanofibers for toxicity studies

**DOI:** 10.1002/1348-9585.12176

**Published:** 2020-11-07

**Authors:** Takafumi Sai, Junko Maru, Sawae Obara, Shigehisa Endoh, Hideo Kajihara, Katsuhide Fujita

**Affiliations:** ^1^ Innovation Promotion Division Oji Holdings Corporation Tokyo Japan; ^2^ Research Institute of Science and Sustainability (RISS) National Institute of Advanced Industrial Science and Technology (AIST) Tsukuba Japan

**Keywords:** cellulose nanofibers, preservatives, sterilization, toxicity study, viscosity

## Abstract

**Objectives:**

The aim of this study is to establish a sterilization method for cellulose nanofibers (CNFs) dispersions that uses multiple preservatives with different hydrophilicities without affecting the physical and chemical properties of CNFs, and to provide useful information for sample preparation in future toxicity study of CNFs.

**Methods:**

Various preservatives were added to the phosphorylated CNF dispersions, endotoxin level and the numbers of bacteria and fungi in the CNF dispersion were analyzed. The pH values and viscosity of sterilized CNF dispersions were compared with those of control and autoclaved CNF dispersions.

**Results:**

Phosphorylated CNF dispersions at a concentration of 2.0 mg/mL or lower and the addition of 10 µg/mL benzalkonium chloride alone or 250 µg/mL methyl parahydroxybenzoate and 250 µg/mL propyl parahydroxybenzoate in combination can sterilize CNF dispersions without changing the physical and chemical properties of CNFs.

**Conclusions:**

We developed sterilization method for CNF dispersions that uses multiple preservatives with different hydrophilicities without affecting the physical and chemical properties of CNFs. This sterilization method for CNFs dispersions can be applied to the safety assessment of CNF with different physicochemical properties in the future.

## 1 INTRODUCTION

Cellulose, the most abundant polymer on Earth, is present in plant cell walls and is used as a raw material for paper.^1^ Cellulose is a carbohydrate consisting of repeats of two glucose units joined by β‐1,4 glycosidic linkages, and cellulose chains constitute crystal structures linked by intermolecular and intramolecular hydrogen bonds.^2^ The most basic unit of cellulose is the cellulose microfibril, which has a width of approximately 3‐4 nm. Microfibril bundles, which have a width of 10‐20 nm, represent a basic unit in the cell wall.^3^ Furthermore, these bundles grow in size to several hundreds of nanometers and form a nested network, thereby comprising microfibrillated cellulose.^4^ Cellulose has long been used in products such as wound‐healing products, dialysis membranes, and food additives.^1^ In recent years, the development of cellulose nanofibrils, also called cellulose nanofibers (CNFs), in which its fibers are dispersed into nanomaterials, which are 100 nm or smaller in at least one dimension, have been progressed. However, little is known about the potential biological effects of CNFs. CNFs contain mechanically defibrated fibers obtained via mechanical fibrillation using a disk mill or chemical modification of the hydroxyl group on the cellulose surface. Chemically modified CNFs are generally produced by chemically modifying the hydroxyl group of glucose, which is a constituent unit of cellulose, and various methods for producing chemically modified CNF have been developed.^5^ One example is phosphorylated CNF in which the hydroxyl groups at the C2, C3, and C6 positions of glucose are replaced with a phosphate group by urea and ammonium dihydrogen phosphate.^6^ Recent research on CNFs having various functions has been advanced by substituting these hydrophilic groups with high molecular weight hydrophobic groups in an organic solvent. Polyethylene glycol, polyethylene imine, a fluoro compounds, etc have been used as high molecular weight groups, and functions, such as antimicrobial property and oil resistance, have been added to CNFs.^5^ Chemically modified CNF has a diameter of 3‐4 nm and a length of over 2000‐3000 nm with high dispersibility.^4^, ^6^ The characteristics of CNFs include a low coefficient of thermal expansion (approximately 2.7 ppm/K) because of their high crystallinity and high light transmittance of cast film (90% or greater) because of their ultrafine characteristics.^7^

CNFs represent a promising new material for various applications such as structural and healthcare materials because of their mechanical properties and adsorptivity.^1^ However, because CNFs are nanomaterials, various toxicity studies have been conducted to assess their potential harm in humans. Because cellulose itself feeds various bacteria and fungi and the manufacturing process is not sterile, CNFs is likely to be contaminated, and sterilization is necessary for CNF toxicity studies. Although a very strong chemical treatment added in the initial production process of CNFs doubles as a sterilizing action, the production equipment needs to be reworked in order to produce the final product without contamination.^8^ However, although a number of in vivo CNF toxicity studies have been conducted, few papers have been experimented after confirming that the physical and chemical properties of CNF samples after treatments such as sterilization have not changed compared to untreated CNF.

Heating, radiation irradiation, or filtration can be mentioned as a candidate method of sterilizing CNF. However, previous studies revealed that some chemically modified CNFs change properties by heating at 165°C for less than few minutes,^6^ and γ‐ray irradiation reduce the degree of cellulose polymerization and tensile strength of fibers by cellulose chain cleavage.^9^ Furthermore, because the pore diameter of the filter for sterilizing filtration is less than 1 µm, it is presumed that the possibility that the CNF fiber is caught by the filter is high. These facts indicate that autoclaving, filtration, and radiation irradiation may not be appropriate methods for CNF sterilization.^10^, ^11^, ^12^

On the other hand, various preservatives are added to the pulp. In the past, oxidizing agents such as hypochlorous acid were used for antiseptic purposes, in recent years, there is a tendency to use organic nitrogen compounds such as hydantoin, sulfamate, isocyanurate, and compounds in which chlorine is attached to these nitrogen compounds.^13^ Although the effect on properties has not been confirmed, a safety study which CNF with preservative organic nitrogen compounds is administered to animals has been reported.^14^ Therefore, although it depends on the kind of chemical substance, it was speculated that CNF may be able to be sterilized without affecting its physical and chemical properties by adding preservatives. This study aimed to confirm the possibility to sterilizing CNFs without influencing their physical and chemical properties for use in toxicity studies.

## 2 MATERIALS AND METHODS

### 2.1 Test materials

A 20 mg/mL phosphorylated CNF slurry (Oji Holdings., Ltd.) was used as the raw material. The CNF sample was stored at 4°C and protected from light for approximately 1 month from production to use. Methyl parahydroxybenzoate (MPHB), propyl parahydroxybenzoate (PPHB), benzalkonium chloride (BAC), and dehydroacetic acid were purchased from Wako Pure Chemical. Methylene bisthiocyanate and distilled water (DW) were purchased from Toronto Research Chemicals and ThermoFisher Scientific, respectively.

### 2.2 Selection of contamination index

Three indicators were used to quantitatively evaluate CNF contamination: (a) the number of bacteria, (b) the number of fungi, and (c) the endotoxin level. Endotoxin is a glycolipid constituting the cell wall of gram‐negative bacteria that causes shock because of the immune response, mainly promoting the secretion of inflammatory cytokines, but it is not eliminated even if the bacteria themselves are sterilized by denaturing the protein with preservatives.^15^

### 2.3 Endotoxin analysis

An Endospecy ES‐50M kit was purchased from Seikagaku Corporation, and 0, 0.01, 0.05, 0.10, and 0.20 EU/mL solutions for calibration were prepared according to the manufacturer's instructions. Fluorescence at wavelengths of 410 and 495 nm was measured using a microplate reader (BIO‐RAD), and a linear response of *R* ^2^ >0.99 was achieved. The 20 mg/mL phosphorylated CNF slurry was diluted 100,000‐fold with DW, and the concentration of endotoxin in the dispersions was measured.

### 2.4 Colony count

Petrifilm AC and YM sheets were purchased from 3M to detect bacteria and fungi, respectively. One milliliter of 2.0 mg/mL phosphorylated CNF was dispensed on Petrifilm AC and YM sheets, which were incubated at 35°C for 2 days and at 22°C for 5 days, respectively, after which the numbers of colonies were counted (n = 1 or 2).

### 2.5 Selection of preservatives

A general antiseptic, not an antibiotic for medical use, was used in this study because CNFs were contaminated by various bacteria and fungi and general antiseptics are available easily. Table 1 shows the preservatives used in this study, the upper limit concentrations of these preservatives added to medical drugs in Japan,^16^, ^17^ their solubility in various solvents,^18^ and the upper limit concentrations of preservatives in the CNF dispersion in this study. In this study, the upper limit concentrations of preservatives added to medical drugs in Japan and those of antiseptic agents added to CNF dispersions in this study were identical.

**TABLE 1:** Preservative candidates for sterilization of phosphorylated CNF

Preservatives	Maximum concentration in medicine (µg/mL)		Solubility (mg/mL) at 25°C		Maximum concentration in this study (µg/mL)
	Inhalant	Ophthalmic	In water	In ether	
MPHB, PPHB	‐	500	2.5 (MPHB), 0.5 (PPHB)	100‐1000	(in total) 500
BAC	100	100	>1000	<0.1	100
Dehydroacetic acid	‐	500	(as Na salt) 330	50	500

### 2.6 Preparation of preservative solutions and dissolution of CNF dispersion

The concentration of each preservative solution was adjusted to 11.1% higher than the final concentration used in the experiment. In particular, aqueous solutions of 555 µg/mL MPHB, 555 µg/mL PPHB, 555 µg/mL dehydroacetic acid, 111 and 11.1 µg/mL BAC, and 278 µg/mL each of MPHB and PPHB (MPHB/PPHB) were prepared. MPHB/PPHB were added to DW and dissolved at 80°C for 30 minutes. Other preservatives were dissolved in DW at room temperature. Phosphorylated CNF dispersions with preservative were prepared by mixing the 20 mg/mL phosphorylated CNF slurry with aqueous solutions of preservatives at a ratio of 1:9 using an ARE‐310 planetary mixer (THINKY) for 60 minutes per 30‐mL dispersion at room temperature. CNF samples for control and autoclave were prepared using DW and ARE‐310 planetary mixer.

### 2.7 Physical and chemical properties of CNF dispersion

A dispersion of 2.0 mg/mL phosphorylated CNF containing 10 µg/mL BAC was diluted with 10 µg/mL BAC aqueous solution to a concentration of 1 or 0.5 mg/mL. Similarly, 2.0 mg/mL phosphorylated CNF dispersion containing 250 µg/mL each of MPHB/PPHB was processed in the same manner to a concentration of 1.0 or 0.5 mg/mL using 250 µg/mL each of MPHB/PPHB. A dispersion containing 2 mg/mL phosphorylated CNF for autoclave was diluted with DW to a concentration of 1.0 or 0.5 mg/mL and autoclaved at 121°C for 20 min (TOMY). The measurements of pH and viscosity were performed after sterilization of the samples was confirmed. The pH of each concentration of phosphorylated CNFs was measured using an F53 pH/ION Meter (Horiba) according to the manufacturer's protocol, and the measurements were repeated three times. The viscosity of the phosphorylated CNF dispersions containing a preservative were measured using an MCR‐302 rheometer (Anton Paar) with cone‐plate geometry (CP‐25‐2) at 25°C in the shear rate range 1‐1000 s^−1^ according to the manufacturer's procedure (n = 3). A drop of the 10 µg/mL CNF dispersion was mounted on a grid, and one drop of uranyl acetate was added as a negative stain for the CNF. Specimens were observed using TEM (FEI Tecnai) at an accelerating voltage of 120 kV. The zeta potential of the 2.0 mg/mL phosphorylated CNF dispersion was measured using a Zetasizer Nano ZS (Malvern Panalytical) (n = 3).

### 2.8 Statistical analysis

Results were expressed as the mean ± standard deviation (SD). Statistical analyses were carried out with the *F*‐test and Student's *t*‐test to assess differences between data groups. Differences were considered significant when *P* < .05.

## 3 RESULTS

### 3.1 Contamination analysis

According to the fluorescence intensity of the 0.2 µg/mL phosphorylated CNF dispersion, the endotoxin concentration was 0.2632 EU/mL (data not shown). Therefore, it was calculated that the 2.0 mg/mL phosphorylated CNF dispersion contained 2632 EU/mL endotoxin. Furthermore, a large number of bacterial colonies (approximately 500 or more) and 102 fungi colonies were detected from 1 mL of the 2.0 mg/mL phosphate‐esterified CNF dispersion.

### 3.2 Sterilization treatments

Table 3 shows the names and concentrations of various preservatives added to the CNF dispersion and whether bacterial colonies were detected 1‐3 weeks after preparation. As shown in Table 1, the added concentrations of these preservatives were below the maximum concentration in medicines, and it was presumed that they did not affect living bodies. The 2.0 mg/mL phosphorylated CNF dispersion was sterilized when MPHB/PPHB was added at a concentration of 250 µg/mL each or when BAC was added at a concentration of 10 µg/mL or more (Table 2). Incidentally, BAC is a surfactant containing chloride ions, which may act on phosphorylated CNF to change its physical and chemical properties. Thus, to minimize the concentration as much as possible, the sample was prepared at 10 µg/mL. To confirm the details of sterilization using MPHB/PPHB, BAC, or autoclave, changes in the number of colonies in relation to time or concentration were evaluated. Sterilization of the CNF dispersion was confirmed after 4 weeks when MPHB/PPHB was added at a concentration of 250 µg/mL. The sample containing 10 µg/mL BAC was sterilized after 1 week, but sterilization could not be confirmed using 4 µg/mL BAC. The autoclaved sample was sterilized (Table 3). No fungi were detected from the CNF dispersion containing 250 µg/mL each of MPHB/PPHB, 10 µg/mL of BAC, and autoclaved (data not shown).

**TABLE 2:** Bactericidal evaluation for selection of preservatives (n = 1)

Preservatives	Concentration (µg/mL)	Weeks after dispersion formulation		
		1	2	3
Dehydroacetic acid	500	No	No	No
Methylene bisthiocyanate	10	No	No	No
MPHB	500	No	No	No
PPHB	500	No	No	No
MPHB/PPHB	250, respectively	No	No	Yes
BAC	10	Yes	‐	‐
BAC	100	Yes	‐	‐

Note

"Yes": detected colonies were less than 5/mL, "No": detected colonies were more than 5/mL.

**TABLE 3:** Colony numbers of bacteria in 0.2% phosphorylated CNF dispersion with preservatives or autoclaved (n = 2)

Preservative or preparation	Concentration (µg/mL)	CNF dispersion without preservative	Weeks after dispersion formulation			
			1	2	3	4
Negative control	0	TNTC, TNTC	‐	‐	‐	‐
MPHB/PPHB	250, respectively	‐	‐	TNTC, 34	169, 18	0, 0
BAC	4	‐	0, 21	‐	‐	‐
BAC	10	‐	0, 0	‐	‐	‐
Autoclave	0	0, 0	‐	‐	‐	‐

Note

"‐": evaluation was not carried out, "TNTC": too numerous to count.

### 3.3 Physical and chemical properties of CNF dispersion

The pH values of 0.5, 1.0, and 2.0 mg/mL phosphorylated CNF dispersions without sterilization treatment, with MPHB/PPHB, with BAC, and with autoclave, respectively, are shown in the Table 4. Because sterilization of the samples took 4 weeks for PPHB and MPHB addition and 1 week for BAC addition, pH and viscosity measurements were performed 4 and 1 weeks after preparation, respectively.

**TABLE 4:** pH values of phosphorylated CNF dispersion with preservatives or autoclaved

Preservative or preparation	Concentration (µg/mL)	Concentration of CNF (mg/mL)		
		0.5	1.0	2.0
Control	0	8.2	8.3	8.4
MPHB/PPHB	250, respectively	8.0	8.2	8.4
BAC	10	8.4	8.6	8.7
Autoclave	0	8.1	7.9	7.7

**FIGURE 1:**
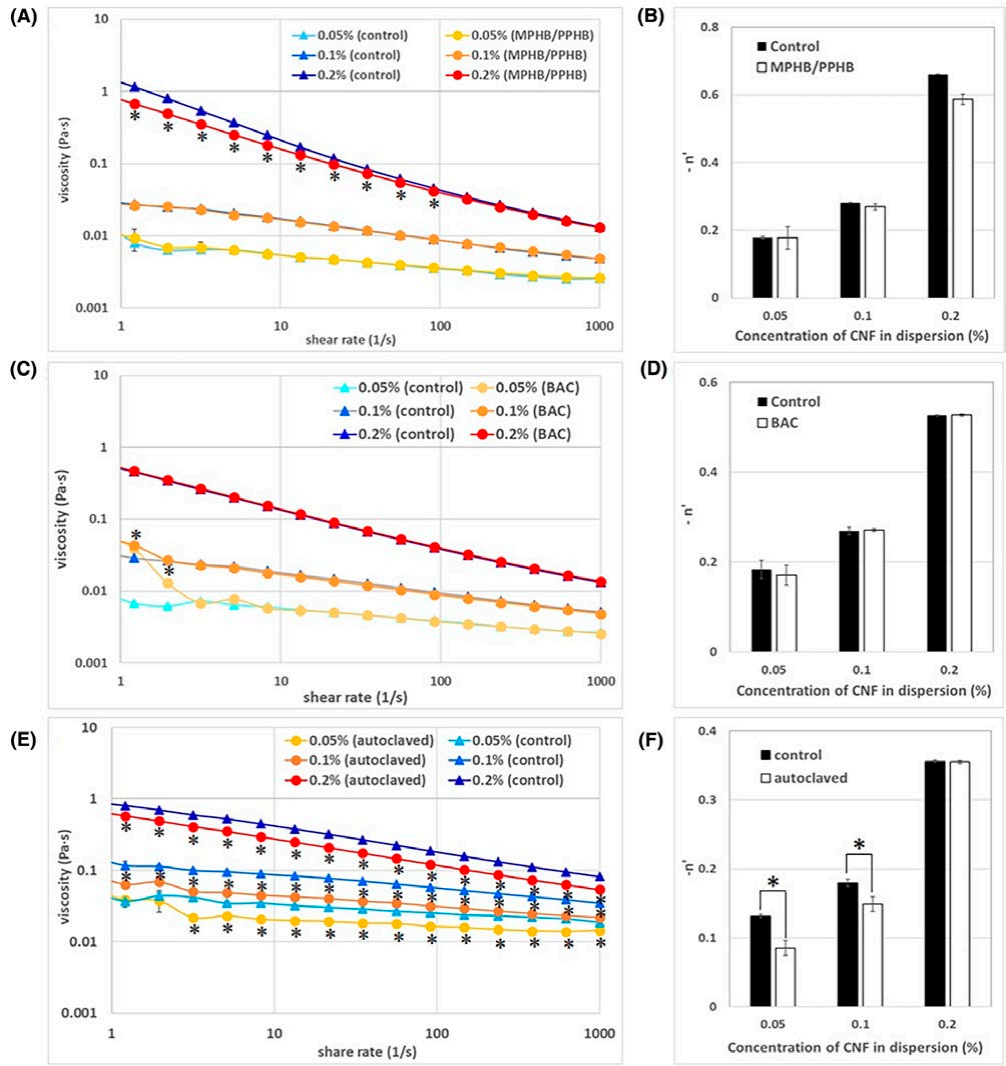
Shear viscosity (A, C, and E) and –n' (B, D, and F) of the phosphorylated CNF dispersions. CNF dispersions containing MPHB/PPHB (A and B), BAC (C and D), and no preservatives (E and F) (n = 3). *: *P* < .05

The viscosities of phosphorylated CNF dispersions at concentrations of 0.5, 1.0, and 2.0 mg/mL, in which MPHB/PPHB was added at a concentration of 250 µg/mL, were measured using a rheometer 4 weeks after preparation, and the viscosity curve is shown in Figure 1A. The viscosity of each sample decreased as the shear rate increased, and the samples behaved as non‐Newtonian liquids. In the 2.0 mg/mL CNF dispersions containing MPHB/PPHB, the viscosity was significantly lower than that of the control CNF dispersion in the low shear rate region, but no difference was observed at shear rates of approximately 100 s^−1^ or higher. The absolute values of the gradient of the flow curves of each concentration of CNF dispersions at shear rates of 3.1‐619 s^−1^ are shown in Figure 1B. Although the absolute values of the gradient of the flow curves were CNF concentration‐dependent, the values were not altered by the presence of MPHB/PPHB.

The viscosities of 0.5, 1.0, and 2.0 mg/mL phosphorylated CNF dispersions containing 10 µg/mL BAC were measured using a rheometer 1 week after preparation, and the viscosity curve is shown in Figure 1C. The viscosity of each sample decreased as the shear rate increased, and the samples behaved as non‐Newtonian liquids. In the 0.5 mg/mL CNF dispersion containing BAC, the viscosity was significantly higher than that of the control CNF dispersion in the low shear rate region, but no difference was observed at higher shear rates. The absolute values of the gradient of the flow curves of each concentration of the CNF dispersions at shear rates of 3.1‐619 s^−1^ are shown in Figure 1D. Although the absolute values of the gradient of the flow curves were CNF concentration‐dependent, no difference was observed in the presence or absence of BAC.

**FIGURE 2:**
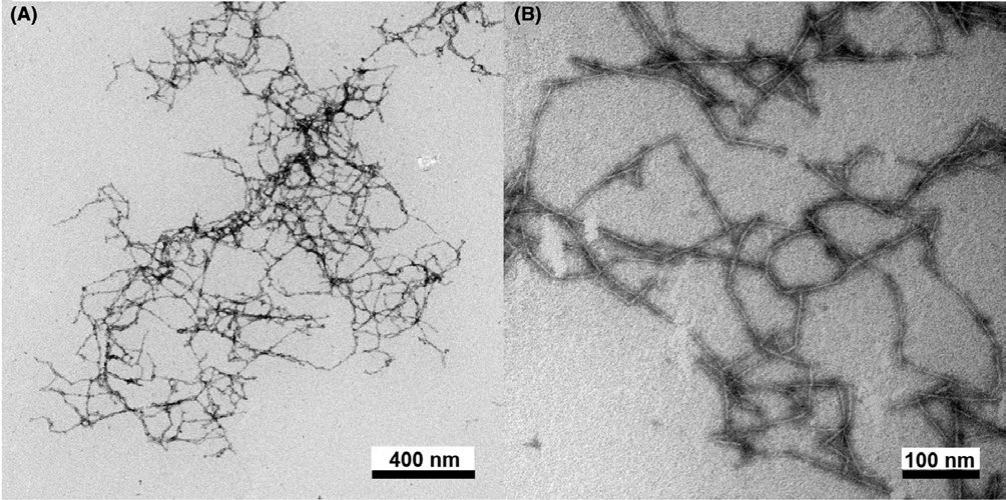
Low (A) and High (B) magnification of TEM images of phosphorylated CNF dispersion without preservative

The viscosities of 0.5, 1.0, and 2.0 mg/mL autoclaved phosphorylated CNF dispersions were measured using a rheometer after preparation, and the viscosity curve is shown in Figure 1E. The viscosity of each sample decreased as the shear rate increased, and the samples behaved as non‐Newtonian liquids. In the 1.0 and 2.0 mg/mL autoclaved CNF dispersions, the viscosity was significantly lower than that of the control CNF dispersion in the all shear rate region. In the 0.5 mg/mL autoclaved CNF dispersion, the viscosity was significantly lower than that of the control CNF dispersion outside the low shear rate region. The absolute values of the gradient of the flow curves for each concentration of the CNF dispersions at shear rates of 3.1‐619 s^−1^ are shown in Figure 1F. Although the absolute values of the gradient of the flow curves were CNF concentration dependent, the values of autoclaved CNF samples were significantly lower in the low concentration samples than in the control samples. TEM images of representative phosphorylated CNF dispersion without preservatives were shown in Figure 2 (A and B are low and high magnification, respectively). Dispersed CNF with a width of 3‐4 nm were observed, and no significant difference from CNF dispersion with preservatives was observed (data not shown). In the zeta‐potential measurement of CNF dispersion, the device warned that the material size in the sample was not uniform. Under these conditions, the zeta potential of 2.0 mg/mL phosphorylated CNF dispersion was −81.2 ± 7.3 mV, and no significant difference from CNF dispersion with preservatives was observed (data not shown).

## 4 DISCUSSION

Because this study aimed to confirm the possibility to sterilizing CNFs without influencing their physical and chemical properties for use in toxicity studies, sterilization using γ‐ray irradiation or filtration was not considered because these methods can affect the physical and chemical properties of CNFs. The results obtained in this study revealed that some preservatives can sterilize CNFs without changing their physical and chemical properties. However, the physical and chemical properties of CNF were changed by the autoclave treatment.

The concentration of the CNF sample used in this study was set assuming that it will be compared with that of carbon nanotubes (CNTs), a novel nanomaterial already in use, in future toxicity studies. Regarding the inhalation toxicity evaluation of CNTs, many intratracheal administration studies using CNT dispersions have been conducted. In a 3‐month study using rodents, non‐transitory inflammatory changes such as chronic inflammation, fibrosis, and increased levels of various cytokines in bronchoalveolar lavage fluid (BALF) with CNT administration at concentrations of 2 mg/kg or higher were observed in the lungs.^19^, ^20^ Therefore, the maximum concentration of the CNF dispersion was set at 2 mg/g to match the concentration of 2‐3 mg/kg in the standard dosage (1.0‐1.5 mL/kg) for rodents in the intratracheal administration study.

Endotoxin was detected in the obtained CNF samples. Based on the measured endotoxin concentrations, the endotoxin exposure levels in the case of inhalation or ingestion of 2.0 mg/mL CNF dispersions administered intratracheally^21^, ^22^ or orally^23^, ^24^ in toxicity studies (1.0‐1.5 mL/kg and 10 mL/kg, respectively) were 2623‐3934 and 26 232 EU/kg, respectively. According to a prior study,^25^ endotoxin concentration of 1 µg/kg or higher originally derived from *E. coli* is required for inflammation in the lungs following intratracheal administration in mice, and this amount corresponds to endotoxin concentrations in the EU/kg range of hundreds of thousands.^26^ In addition, it is generally known that endotoxin does not affect the gastrointestinal tract, and in the literature, oral administration of 1 million EU/kg endotoxin had no gastrointestinal effects. Therefore, although endotoxin levels are not decreased by sterilization using preservatives, it is presumed that the possibility of harm to living bodies is low depending on the amount of endotoxin present in the CNF samples used in this study. Because endotoxin is an extremely stable molecule that is resistant to extreme temperature and pH compared with protein,^15^ to remove or inactivate endotoxin, ion exchange chromatography, ultrafiltration, or dry heat sterilization must be performed.^27^, ^28^, ^29^ However, these methods are inappropriate for toxicity studies because they are likely to result in denaturation of chemically modified CNFs, making it necessary to measure the endotoxin concentration of the samples. It is possible to refer to the improvement of the process for the production of ecdotoxin‐free CNF suggested by previous study.^8^

The CNF dispersions could not be sterilized even if 500 µg/mL MPHB or PPHB alone was added, but by adding 250 µg/mL of both MPHB and PPHB, it was possible to sterilize the CNF dispersion without changing the total concentration of additives. In the case of esters containing MPHB/PPHB, synergistic improvement of antibacterial ability has been observed, and this property is also frequently used in the preparation of pharmaceuticals for patients.^30^ It was inferred that this synergistic effect was the one reason why only the sample to which MPHB/PPHB were added in combination was sterilized in this study. MPHB/PPHB and BAC have bacteriostatic^31^ and bactericidal effects,^32^ respectively. The time required to sterilize the CNF dispersions was 4 weeks for MPHB/PPHB and 1 week for BAC, but this difference could be attributable to the difference in the mechanism of action between MPHB/PPHB and BAC. CNF dispersion was also sterilized via autoclaving.

The pH of the CNF dispersions at each concentration ranged 8.0‐8.7. For CNF dispersions lacking preservatives, the pH of the 20 mg/mL dispersion (9.3, data not shown) was 0.9 higher than that of the 2.0 mg/mL dispersion, and CNF concentration‐dependent increases in the pH were confirmed in the range 0.5‐2.0 mg/mL. The pH of the CNF dispersions was changed by no more than 0.2 relative to the control value after the addition of MPHB/PPHB. Meanwhile, the addition of BAC increased the pH by 0.2‐0.3, in line with the addition of MPHB/PPHB. However, in the autoclaved CNF sample, a concentration‐dependent decrease in pH value was observed. In phosphorylated CNF, because the OH group is substituted with a phosphate group, the phosphate group was removed by heating and pressurization by autoclave treatment, and it was speculated that the generation of phosphoric acid was the cause of the decrease in pH value. Furthermore, it was inferred that the degree of pH value decrease is proportional to the concentration, that is, the amount of phosphoric acid. In general, it is known that chemical burns caused by basic substances progress over a long period,^33^, ^34^ but serious changes, including liquefactive necrosis of tissues, occur at pH values of 11.5 or higher.^35^, ^36^ Based on the measured pH, the CNFs used in this study have a low possibility of causing chemical burn injury in living tissue, whereas depending on the additives or modifying group of CNFs, the pH of the sample may be affected, permitting even substances close to neutrality to cause chemical burns with prolonged exposure.^37^

Regarding CNF dispersions containing preservatives, the viscosity differed from that of the control dispersions only for low share rates, but when the shear rate was about 100 s^‐1^ or more, no significant difference was observed. In processes such as mixing, stirring, spraying, and injection, a shear rate of 100 s^−1^ or higher is applied to the dispersion. Therefore, it was inferred that the physical and chemical properties of the CNF dispersion without influenced by the addition of the preservative could be obtained in the preparation of the CNF dispersion. In the autoclaved CNF sample, the viscosity decreased in the range 1‐1000 s^−1^ in almost all samples compared with the control sample, and a significant decrease in the ‐n' value was observed in the low concentration sample. It was concluded that the sample having a smaller number of cellulose fibers in the CNF dispersion was more susceptible to heating and pressing.

This study aimed to confirm the possibility to sterilizing CNFs without affecting their physical and chemical properties for use in toxicity studies. Using phosphorylated CNF, sterilization of CNF dispersions at concentrations of 2.0 mg/mL or lower could be achieved by adding 10 µg/mL BAC or 250 µg/mL MPHB/PPHB without changing their pH and viscosity. It is known that BAC has high solubility in water,^38^ whereas MPHB/PPHB are more soluble in organic solvents such as ether.^39^, ^40^ At present, CNFs having characteristic properties are being developed via various chemical modifications. For hydrophobic CNFs, water may be inappropriate as a dispersion medium, and depending on the application, it is necessary to conduct a toxicity study. However, in the autoclaved CNF, changes in the physical and chemical properties, which considered to be the effects on the chemical modification group were observed. Because the autoclave exerts a strong force, it is considered to be unsuitable as a sterilization method depending on the property of the chemical modification group of CNF.

## 5 CONCLUSIONS

This study, which found that CNF dispersions can be sterilized without affecting their physical and chemical properties using either water‐soluble BAC or organic solvent‐soluble MPHB/PPHB will broaden the options of preservatives used for toxicity studies of CNFs with various physical and chemical properties. It is expected that research and development of CNFs with various properties will be proceeded in the future. However, since CNF is a new nanomaterial, there is a concern that it will affect human health. Therefore, it is very important to evaluate the safety of each type of CNF using animals. This study, which enabled sterilization of CNF dispersions that use preservatives without affecting the physical and chemical properties of CNFs, provided a first step toward the safety evaluation of various CNFs in the future.

## DISCLOSURE

*Approval of the research protocol*: N/A. *Informed consent*: N/A. *Registry and the registration no. of the study/trial*: N/A. *Animal studies*: N/A. *Conflict of interest*: The authors declare that they have no competing interests.

## AUTHOR CONTRIBUTIONS

TS and KF conceived the ideas; TS, JM, SO, and SE collected the data; TS and SE analyzed the data; and TS, HK, and KF led the writing;
